# Tumeur fibreuse solitaire parautérine: à propos d’un cas

**DOI:** 10.11604/pamj.2016.25.180.8184

**Published:** 2016-11-21

**Authors:** Olfa Slimani, Cyrine Belghith, Sarrah Saoudi, Makhlouf Tahar, Riadh Ben Temim, Nabil Mathlouthi, Leila Attia

**Affiliations:** 1 Service de Gynécologie-Obstétrique A, Hôpital Charles Nicolle, Tunis, Tunisie; 2 Faculté de Médecine de Tunis, Tunisie

**Keywords:** Tumeur fibreuse solitaire parautérine, diagnostic, tractus génital, Parauterine solitary fibrous tumor, diagnosis, genital tract

## Abstract

Les tumeurs fibreuses solitaires du tractus génital féminin sont extrêmement rares. Nous rapportons le cas d’une patiente âgée de 78 ans qui a présenté une masse pelvienne. L’exploration chirurgicale a montré une tumeur parautérine. L’examen anatomopathologique a conclu à une tumeur fibreuse solitaire avec des signes de malignité. Les suites ont été marquées par le décès de la patiente. Il est important de connaître ces tumeurs dont l’évolution peut être péjorative. Un suivi au long cours doit être recommandé pour les tumeurs résécables.

## Abstract (en)

Solitary fibrous tumors of the female genital tract are extremely rare. We report the case of a 78-year old patient presenting with a pelvic mass. Surgical exploration showed parauterine tumor. Anatomo-pathological examination confirmed the diagnosis of malignant solitary fibrous tumor. The evolution was marked by the death of the patient. It is important to be able to detect these tumors whose evolution can be poor. Long-term follow-up of patients with resectable tumor should be recommended.

## Introduction

Les tumeurs fibreuses solitaires (TFS) sont des tumeurs mésenchymateuses rares, longtemps observée au niveau de la plèvre et maintenant elles sont décrites dans de nombreux tissus et organes. Cette pathologie inhabituelle est de diagnostic anatomopathologique et amène à discuter les autres tumeurs des tissus mous. Nous rapportons le cas d’une patiente qui présentait une TFS parautérine. La localisation parautérine reste exceptionnelle. Dans cette topographie, nous rapportons une observation de TFS, qui pose le problème de son diagnostic et de sa prise en charge.

## Patient et observation

**Figure 1:**
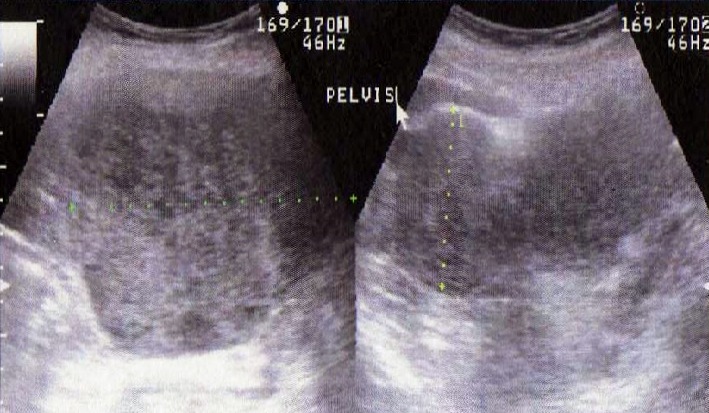
Masse pelvienne polylobée

**Figure 2:**
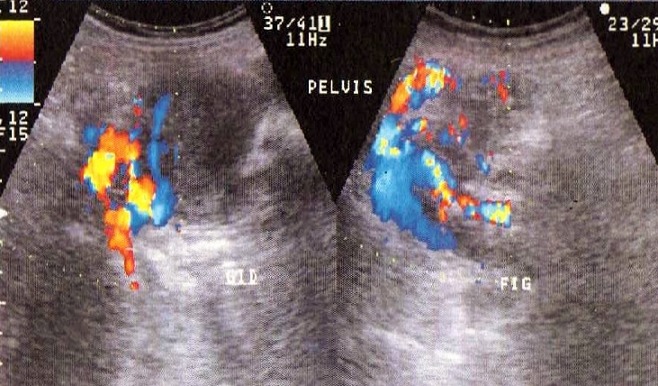
Hypervascularisation de la masse avec hypervascularisation iliaque bilatérale

**Figure 3:**
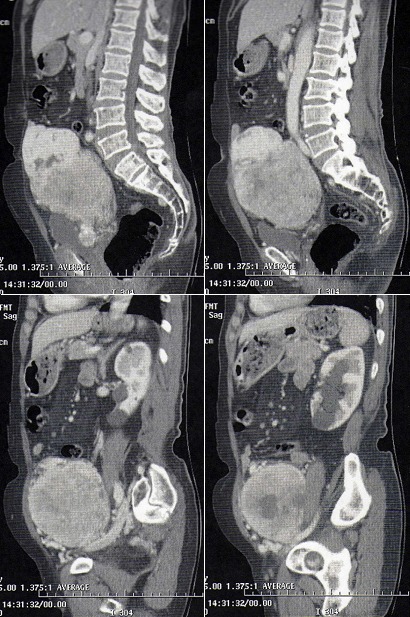
Volumineuse masse du pelvis, intra-péritonéale très hétérogène

**Figure 4:**
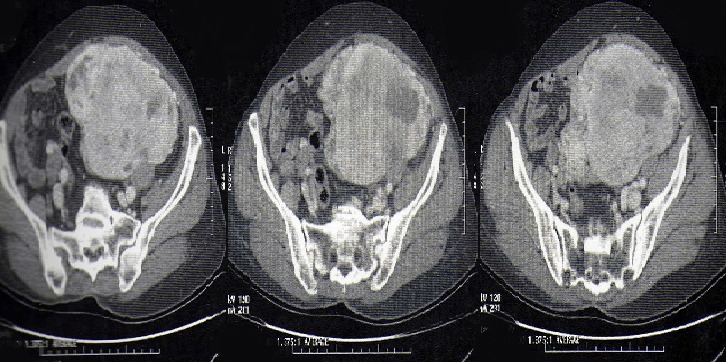
Masse polylobée se rehaussant intensément après injection de produit de contraste avec des zones centrales multiples hypodenses en rapport avec de la nécrose

Il s’agissait d’une patiente âgée de 78 ans, sans antécédent pathologiques notable. La patiente a eu sept accouchements par voie basse sans complications notables et une ligature des trompes il ya 35ans. Elle est ménopausée depuis 25ans. Elle s’était présentée en consultation devant la découverte récente d’une masse pelvienne avec des algies pelviennes évoluant depuis une année, négligées initialement. L’examen physique notait la présence d’une masse pelvi-abdominale s’étalant de la fosse iliaque droite à la fosse iliaque gauche à surface régulière, au dessus de la symphyse pubienne mesurant 20cm de diamétre. L’examen des seins était sans anomalie. L’examen au speculum montrait un col macroscopiquement sain qui a perdu son relief. Au toucher vaginal, l’utérus était difficile à apprécier avec perception d’une masse sus utérine, arrivant à l’ombilic, ne comblant pas les culs de sac. Le reste de l’examen somatique était sans particularité et les aires ganglionnaires étaient libres. Une échographie abdomino-pelvienne, indiquée pour étayer le diagnostic a montré un utérus de petite taille à paroi calcifiée, la présence d’une masse polylobée composée de trois formations de structures tissulaires mesurant 5cm, 7,5cm et 10cm, une hypervascularisation iliaque bilatérales et les ovaires ne sont pas franchement individualisables (Figure 1, Figure 2). Nous avons complété notre exploration par la réalisation d’un scanner abdomino-pelvien qui a montré la présence d’une volumineuse masse du pelvis, intra-péritonéale très hétérogène, à contours bien limités, mesurant au moins 15 cm sur son plus grand axe polylobée se rehaussant intensément après injection de produit de contraste avec des zones centrales multiples hypodenses en rapport avec de la nécrose. La masse est localisée surtout au niveau de la face interne des vaisseaux iliaques au sein des deux loges ovariennes. Il existe aussi un refoulement de la vessie qui est comprimée et refoulée à droite et une circulation veineuse collatérale pelvienne par compression des chaines iliaques et de tout le petit bassin. Il n’y avait pas de thrombus visible et paradoxalement il n’y avait pas d’adénomégalie significative (Figure 3, Figure 4).

Devant ces constatations cliniques et radiologiques : trois diagnostics ont été évoqués : un sacome utérin, une tumeur ovarienne ou un syndrome de krukenberg. La décision du staff était de réaliser une IRM pelvienne qui a montré deux masses pelviennes vraisemblablement ovariennes droite et gauche. Un syndrome de krukenberg était évoqué en premier lieu. La fibroscopie digestive et la coloscopie étaient sans anomalie. Les marqueurs tumoraux (alpha foeto-protéine, antigène carcino-embryonnaire et CA-125) étaient tous négatifs. Une laparotomie exploratrice a été finalement décidée: à l’exploration, l’utérus était de petite taille atrophique, l’annexe droite était sans anomalie. On a noté la présence d’une masse hypervasularisée faisant 15cm prenant naissance au niveau de la corne gauche incluse dans le ligament large et envahissant le muscle droit, fixée à l’annexe gauche et fusant vers le rétro-péritoine et comprimant les vaisseaux iliaques gauches. La trompe et l’ovaire gauche étaient sans anomalies mais fixés à la tumeur. Nous avons pratiqué une hystérectomie totale avec annexectomie bilatérale et une exérèse de la tumeur. L’examen extemporané a conclu à une tumeur à cellules bizarres fusiformes. On décide de se limiter à ces gestes vue l’état fragilisé de la patiente en peropératoire et en attendant l’étude anatomo-pathologique définitive. L’examen anatomopathologique a conclu à une tumeur fibreuse solitaire avec des signes de malignité : taille importante, présence focale de nécrose tumorale, de densité et de pléomorphisme cellulaires et mitoses fréquentes. L’utérus et les annexes étaient sans anomalies. Les suites opératoires ont été marquées par le transfert de la patiente au service de réanimation suite à une embolie pulmonaire post opératoire. La patiente est décédée après 10 jours de l’intervention.

## Discussion

Les tumeurs fibreuses solitaires (TFS) sont des tumeurs mésenchymateuses qui se développent à partir de tissus conjonctif, et plus particulièrement à partir de la plèvre [1]. D’autres sites peuvent être atteints, comme le péritoine, la thyroïde, les glandes salivaires, le périoste, le tissu sous cutané ou le rétro péritoine [2]. Des centaines de localisations extra-thoraciques ont été décrites à ce jour parmi lesquelles les localisations concernant le tractus génital de la femme qui sont très rares [3, 4]. Les circonstances de découverte de ces tumeurs sont variables. L'hypoglycémie peut être révélatrice dans 10% des cas, et celle ci serait liée à la production par les cellules tumorales de l'insuline-like growth factor [5]. L'échographie montre dans la plupart des cas une masse solide échogène, avec vascularisation importante au doppler. Le scanner montre une masse ayant une densité similaire au muscle avec des calcifications. Après injection de produit de contraste, la tumeur prend le produit de contraste sauf quelques plages liquidiennes pouvant évoquer une nécrose tumorale. L'IRM permet après injection de bolus de 2ml/s de gadolinium de montrer une masse iso intense au muscle en T1 et un signal hétérogène plutôt hypo intense en T2. Ainsi, Le diagnostic des tumeurs fibreuses solitaires est difficile du fait de leur localisation inhabituelle et de la variabilité du profil anatomopathologique. Dans notre observation, le diagnostic était difficile et c’est l’étude anatomopathologique qui a conclu à une tumeur fibreuse solitaire.

L’examen histologique d’une TFD montre une tumeur ferme de couleur claire (beige rosée ou grise) à centre fibreux, composée de cellules fusiformes de disposition hétérogène. Les mitoses sont rares (moins de 4 mitoses pour 10 champs à fort grossissement). Il s’agit de tumeurs qui alternent hypocellularité et hypercellularité, avec des bandes épaisses de fibrose hyaline. Les cellules peuvent être rondes ou fusiformes. L’étude immuno histochimique est une aide précieuse au diagnostic positif. La majorité des TFS sont positives pour le CD34 [6–8] et pour le bcl-2 [9, 10]. Elles sont en général négatives pour les cytokératines, l’actine musculaire lisse, la desmine, la protéine S-100 et le c-kit [11]. Le seul traitement qui soit efficace est chirurgical. Compte tenu du risque élevé de récidive locale, une exérèse complète s’impose [6]. Il n’existe aucun consensus concernant les marges anatomopathologiques lors de la résection chirurgicale, cependant celle-ci doit être maximale(R0). On pourrait proposer une prise en charge thérapeutique similaire à celle des sarcomes, qui propose un examen extemporané au niveau des berges de la tumeur. Cela permet une résection maximale pour la tumeur et minimale pour les tissus adjacents sains. Liu et al. dans une récente étude sur les sarcomes des parties molles proposent une résection chirurgicale avec une marge de sécurité à 10 mm [12]. Récemment une étude in vitro, testant la sensibilité d'une tumeur fibreuse solitaire avec un index mitotique de 7/50 HPF avec une chimiothérapie. La tumeur était sensible aux 5-FU, adramycine, mitomycine-c et le doctaxel, ceci pourrait ouvrir les portes probablement pour les tumeurs fibreuses solitaires les plus agressives, récidivantes inextirpables ou ayant une exérèse incomplète [13].

L’évolution de ces TFS est souvent lente et indolore, sauf dans le cas de tumeurs volumineuses qui pourraient entraîner des compressions diverses. Dans notre cas, il existait une compression importante des vaisseaux iliaques. Malgré que la plupart des TFS soient bénignes, l'évolution reste imprévisible. Les TFS sont agressives dans 10 à 15% voire malignes avec survenue de métastases pulmonaires, hépatiques et osseuses. La caractéristique principale des TFS est une agressivité locale, même si des évolutions métastatiques tardives ont été décrites. Ces lésions doivent donc être considérées comme potentiellement malignes [6]. Pour notre patiente, L’examen anatomopathologique a conclu à une tumeur fibreuse solitaire avec des signes de malignité. Les récidives locales surviennent suite à une chirurgie d’exérèse incomplète et peuvent survenir tardivement (jusqu’à 9 ans après le diagnostic initial) [6]. Le comportement clinique n'est pas toujours prévisible à partir des constatations morphologiques, mais une hypercellularité, un index mitotique élevé et un aspect anaplasique sont des facteurs de mauvais pronostic. L'extirpabilité est le seul et plus important facteur déterminant de l'évolution [14]. La surveillance clinique et radiographique (radiographie thoracique, scanner abdominal) doit donc être prolongée, mais il n’existe pas à ce jour de consensus clairement établi. Le pronostic de ces TFS de localisation gynécologique est variable. Biedrzycki et al. décrivent neuf cas de TFS de localisation gynécologique : un cas de trompe, un cas de tumeur paraovarienne, un cas de localisation utérine, deux cas de tumeur vaginale et quatre cas de localisation vulvaire [14]. Notre cas porte sur une tumeur fibreuse solitaire parautérine.

## Conclusion

Les TFS du tractus génital féminin sont extrêmement rares. Comme dans les autres localisations la plupart sont classées bénignes et l’évolution est favorable. Cependant, il existe des cas d’évolution maligne pouvant être corrélée à des critères histologiques péjoratifs. Aucun consensus n’existe actuellement concernant ces TFS de localisation gynécologique, mais la prise en charge doit être maximale et multidisciplinaire.

## References

[1] Wang J, Arber DA, Frankel K, Weiss LM (2001). Large solitary fibrous tumor of the kidney: report of two cases and review of the literature. Am J Surg Pathol..

[2] Hasegawat, Matsuno Y, Shimodat, Hasegawaf, Sano T, Hirohaschi S (1999). Extra thoracic solitary fibrous tumors: Their histological variability and potentially aggressive behavior. Hum Pathol..

[3] Biedrzycki OJ, Singh N, Habeeb H, Wathen N, Faruqi A (2007). Solitary fibrous tumor of the female genital tract a case report and review of the literature. Int J Gynecol Pathol..

[4] Enzinger Weiss (2001). Soft tissue tumours..

[5] Adhami N, Ahmed R, Lento P, Shimshi M, Herman S, Teirstein A (2004). Fibrous pleural tumor with hypoglycaemia: case study. Mount Sinai J Med..

[6] Vallat-Decouvelaere AV, Dry SM, Fletcher CD (1998). Atypical and malignant solitary fibrous tumors in extrathoracic locations: evidence of their comparability to intra-thoracic tumors. Am J Surg Pathol..

[7] Fukunaga M, Naganuma H, Ushigome S, Endo Y, Ishikawa E (1996). Malignant solitary fibrous tumour of the peritoneum. Histopathology..

[8] Aiba S, Tabata N, Ishii H, Ootani H, Tagami H (1992). Dermatofibrosarcoma protuberans is a unique fibrohistiocytic tumour expressing CD34. Br J Dermatol..

[9] Fukunaga M (2000). Atypical solitary fibrous tumor of the vulva. Int J Gynecol Pathol..

[10] Hasegawa T, Matsuno Y, Shimoda T, Hirohashi S, Hirose T, Sano T (1998). Frequent expression of bcl-2 protein in solitary fibrous tumors. Jpn J Clin Oncol..

[11] Hasegawa T, Hirose T, Seki K, Yang P, Sano T (1996). Solitary fibrous tumor of the soft tissue: an immunohistochemical and ultrastructural study. Am J Clin Pathol..

[12] Liu CY, Yen CC, Chen WM, Chen TH, Chen PC, Wu HT (2010). Soft tissue sarcoma of extremities: the prognostic significance of adequate surgical margins in primary operation and reoperation after recurrence. Ann Sur Oncol..

[13] Yoshimasu T, Oura S, Hirai I, Kokawa Y, Nishida M, Sasaki R, Kawago M, Yzaki M, Tanino H, Sakurai T, Okamura Y (2004). Histoculture drug response assay for solitary fibrous tumor: a case report. Gan To Kagaku Ryoho..

[14] Bugel H, Gobet F, Baron M, Pfister CH, Sibert L, Grise P (2003). Tumeur fibreuse solitaire du rein et autres localisations à l'appareil urogénital: caracteristiques morphologiques et immunohistochimiques. Prog Urol..

